# scViewer: An Interactive Single-Cell Gene Expression Visualization Tool

**DOI:** 10.3390/cells12111489

**Published:** 2023-05-27

**Authors:** Abhijeet R. Patil, Gaurav Kumar, Huanyu Zhou, Liling Warren

**Affiliations:** Global Statistical and Data Sciences, Teva Pharmaceuticals, West Chester, PA 19380, USA

**Keywords:** single-cell RNA sequencing, scRNA-seq, R Shiny, bioinformatics, gene expression, co-expression, differential expression analysis

## Abstract

Single-cell RNA sequencing (scRNA-seq) is an attractive technology for researchers to gain valuable insights into the cellular processes and cell type diversity present in all tissues. The data generated by the scRNA-seq experiment are high-dimensional and complex in nature. Several tools are now available to analyze the raw scRNA-seq data from public databases; however, simple and easy-to-explore single-cell gene expression visualization tools focusing on differential expression and co-expression are lacking. Here, we present scViewer, an interactive graphical user interface (GUI) R/Shiny application designed to facilitate the visualization of scRNA-seq gene expression data. With the processed Seurat RDS object as input, scViewer utilizes several statistical approaches to provide detailed information on the loaded scRNA-seq experiment and generates publication-ready plots. The major functionalities of scViewer include exploring cell-type-specific gene expression, co-expression analysis of two genes, and differential expression analysis with different biological conditions considering both cell-level and subject-level variations using negative binomial mixed modeling. We utilized a publicly available dataset (brain cells from a study of Alzheimer’s disease to demonstrate the utility of our tool. scViewer can be downloaded from GitHub as a Shiny app with local installation. Overall, scViewer is a user-friendly application that will allow researchers to visualize and interpret the scRNA-seq data efficiently for multi-condition comparison by performing gene-level differential expression and co-expression analysis on the fly. Considering the functionalities of this Shiny app, scViewer can be a great resource for collaboration between bioinformaticians and wet lab scientists for faster data visualizations.

## 1. Introduction

scRNA-seq has emerged as a powerful technology for researchers to investigate diverse biological functions by capturing gene expression at the cellular level to gain valuable insights into the diversity present in all tissues. Many tools have been developed recently for analyzing and visualizing scRNA-seq data. Lewsey et al. [1] developed a single-cell application called scCloudMine that shows expression levels in clusters. This application is designed for plant scientists and focuses on the visualization of user-processed scRNA-seq data using a commercial Microsoft Azure cloud-based platform. Jagla et al. [2] designed SCHNAPPs to explore and interpret scRNA-seq data and associated annotations. The SCHNAPPs application follows workflows from Seurat [3] or Scran [4] packages to perform quality control steps, normalization, clustering, and differential expression analysis. The application processes the raw data and provides visualizations for exploring each of the processing steps. However, the raw single-cell data are complex, and the authors strongly recommended the external validation of statistical findings. Ouyang et al. [5] proposed ShinyCell, which dynamically converts scRNA-seq datasets into interactive interfaces that can be easily explored and shared. The differential expression analysis was not included in this tool because of its long processing time, especially with a large number of cells. Additionally, several tools, such as ASAP [6], Cerebro [7], singlecellVR [8], and CellexalVR [9] support enhanced visualization of single-cell clusters by using either 3D plots or a virtual reality platform. However, these tools do not perform gene differential or co-expression analysis. Ouyang et al. [5] and Cakir et al. [10] conducted a comprehensive comparison of similar Shiny tools such as cellxgene [11], iSEE [12], Loom viewer, SCope [13], Single Cell Explorer [14], UCSC single-cell browser [15], ASAP [6], scSVA [16], and SPRING [17] and described key functionalities that were present or missing in each of these single-cell applications. Among them, ASAP, scSVA, and SPRING are the few tools that support single-cell data processing, while the rest are for visualization purposes. The visualization tools such as iSEE, Loom viewer, Single Cell Explorer, and UCSC single-cell browser do not provide either differential expression or co-expression analysis. Some visualization tools, such as cellxgene and ASAP, provide differential expression but not co-expression analysis. The ShinyCell visualization tool offers co-expression analysis, but the differential expression component is missing due to the long processing time required. Overall, most of these existing tools were designed to perform scRNA-seq analysis and provide limited visualization functions. In our experience, cell-type-specific differential gene expression incorporating both cell- and subject-level variability and co-expression are the key functionalities in a single-cell analysis. To the best of our knowledge, none of the aforementioned tools provides a flexible platform for performing and visualizing differential and co-expression analysis locally through a browser.

To address this problem, we have developed a user-friendly R Shiny app named scViewer that can visualize processed scRNA-seq data and perform comprehensive analyses that include cell-type-specific differential and co-expression analysis of genes where the results can be visualized and interpreted. The differential expression analysis performed at the gene level allows faster computation and provides cell-type-specific statistical significance for the gene of interest using negative binomial gamma mixed modeling (NBGMM) to compare gene expression in individual subjects between groups. Additionally, scViewer provides counts of co-expressing cells for a pair of genes, cell-type-specific expression levels of these genes, and correlations of these genes among the co-expressing cells. To illustrate how our app works, we provide an example on how the data can be processed using our customized pipeline and also provide a processed demo dataset to explore the various features of the app. The comprehensive analytical features in the scViewer app will be of broad interest to the scientific community for analyzing scRNA-seq data.

## 2. Materials and Methods

### 2.1. Infrastructure

scViewer was written in R v4.1.2 programming language (R Foundation, Indianapolis, IN, USA) [18], and the interactive client-side web application was created using Shiny v1.7.2 [19], shinythemes v1.2.0 [20], and shinyjs v2.1.0 [21] Shiny environment packages. Seurat v4.1.1 [3] was used for analyzing scRNA-seq data. SingleCellExperiment v1.16.0 [22] and scater v1.22.0 [23] were used to calculate library size. Nebula v1.2.2 [24] was used for performing differential expression analysis. Data wrangling was performed using the dplyr v1.0.9 [25], stringr v1.4.0 [26], stringi v1.7.6 [27], tidyverse v1.3.1 [28], and DT v0.23 [29] packages. Visualization plots were generated using the ggplot2 3.3.6 [30], cowplot v1.1.1 [31], gridExtra v2.3 [32], and ggpubr v0.4.0 [33] R packages. scViewer is supported by 64-bit machines, including Windows, macOS, and Linux platforms. Its dependencies such as R language and R packages are pre-compiled and loaded into the scViewer application. The deployment does not require any installation and only needs a web browser for users to launch the application locally. The computational time required for analyzing the single-cell data depends on the dimension of the processed data as the computational burden increases exponentially with the number of cells. scViewer is a client-side desktop application, and its speed and performance will vary depending on the hardware configuration of the client’s computer.

### 2.2. Data Input

To demonstrate the functionality and usability of scViewer, we used the publicly available transcriptomic dataset (GSE157827) that was generated from the prefrontal cortical samples of Alzheimer’s disease (AD) patients and normal control (Normal) samples by Lau et al. [34]. For an easy and quick demonstration of the scViewer app, we included a subset of the above dataset with 500 cells in the app.

### 2.3. Data Analysis

**Figure 1:**
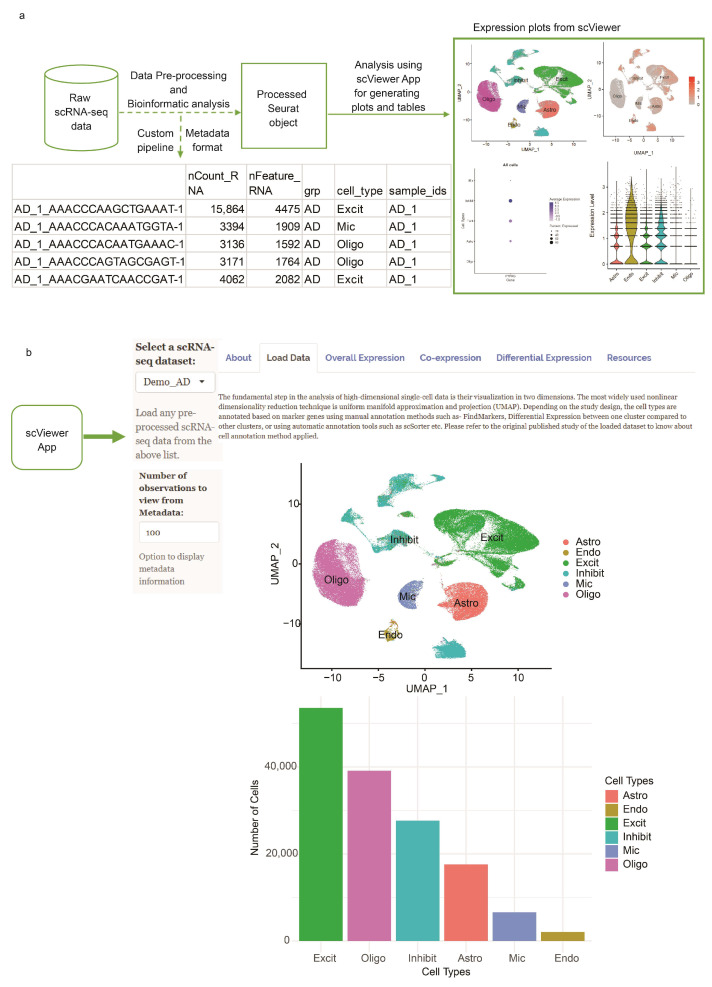
Schematic workflow of the single-cell app, scViewer. (**a**) In the first part, optionally, the raw data can be processed and analyzed following our custom Seurat-based pipeline and the metadata format compatible with scViewer (dotted lines). Next, the users can use the processed Seurat object, and the scViewer app generates various plots and figures. (**b**) Screenshots showing the overview of the scViewer application.

The general workflow for processing the scRNA-seq data starts from the raw count matrix to the generation of a processed Seurat object. Subsequently, the processed Seurat object can be used to perform various downstream tasks, as shown in Figure 1a.

Currently, there is no efficient pipeline that can be used for the automatic processing of scRNA-seq data because this largely depends on the study design and tissue type, as the cell type annotation for different tissues varies. However, a few guidelines are provided by Seurat that can be used to process the data. Following these guidelines, we provide the R code, supporting documentation, and metadata format for processing the raw count matrix to generate the single-cell data object (https://github.com/arpatil01/scViewer, accessed on 25 June 2023). We additionally incorporated the most popularly used single-cell tools for various tasks, including cell annotation. Users can download the scViewer GitHub repository and optionally refer to Supplementary File S1 (https://github.com/arpatil01/scViewer, accessed on 25 June 2023) vignette for generating the Seurat object.

The scViewer app takes the processed Seurat object (.RDS) as input and generates various plots and tables. The complete overview of scViewer is shown in Figure 1a. If users choose to use different data formats, such as Scanpy (AnnData), they can easily convert the objects through conversion functions available in the Seurat framework (https://satijalab.org/seurat/archive/v2.4/conversion_vignette.html, accessed on 25 June 2023).

### 2.4. User Interface

We developed a user interface, as shown in Figure 1b. Users can start by uploading the single-cell dataset of their choice using the drop-down menu, and the corresponding Uniform Manifold Approximation and Projection (UMAP), cell type distribution, and metadata information for the loaded dataset will be displayed. Once the data are loaded successfully, various downstream analyses can be performed in the subsequent tabs.

In the overall expression tab, users can search for a gene of their interest to view the average gene expression across all the cells in the uploaded dataset through various plots such as feature plot, violin plot, and dot plot. These plots show the expression profile of a gene across all the cells from the identified cell types regardless of disease status. In addition, the metrics, including the average expression and percent of cells expressing the gene, are provided. These measurements provide an overview of the expression of the gene across cell types in the loaded dataset.

In the co-expression tab, first, the co-expression of two genes are measured simultaneously and feature plots and tables showing the count and percentage of cells expressing the genes are displayed. Next, based on the user-defined threshold, the subset of cells co-expressing both genes are shown, and violin plots are displayed where the expression level of these genes can be evaluated across different cell types. Lastly, the correlation of expression between these genes in co-expressing cells are computed and the results are plotted using scatterplots across different cell types. In summary, this tab allows users to comprehensively review the expression levels of two genes in the same cells in groups of patients or Normal samples.

In the differential expression tab, the app comprehensively compares gene expression levels and the percentage of cells expressing a particular gene of interest in the patient and Normal groups. Here, we provide the feature, violin, and dot plots for the cells from the patient and Normal groups. We included custom functions in the app to further provide a table displaying the percent expressed and average expression of a gene across disease and Normal groups. Next, we used the NBGMM incorporated in the nebula package to compute the differential expression analysis. The NBGMM accounts for both the subject-level and cell-level overdispersions, but the calculations are computationally intensive [24,35]. Our custom function incorporates NBGMM from nebula [24] which has made NBGMM fast and robust for large-scale single-cell datasets. Further, our analysis was performed at the individual gene level, which can provide gene-specific results on the fly. In our analysis, the differential expression was performed using the raw counts and the corresponding cell library size calculated using scater package [23], which are passed as an input matrix and offset respectively to the nebula’s NBGMM method. The differential expression results are reported using the *p*-value and log_2_FoldChange (log_2_FC). Lastly, we performed pseudobulk analysis where the normalized average expression of a gene across all the cells in each subject was computed and is shown using boxplots.

## 3. Results

In order to illustrate the use of the scViewer app, we used the single-cell expression profiling data of the prefrontal cortex from postmortem human brain of 12 Alzheimer’s disease (AD) patients and 9 normal control (Normal) samples from Lau et al. [34]. We used all 21 samples from the AD and Normal groups for the comparison in our study. We first obtained the raw dataset from GSE157827 and processed the data using our customized Seurat pipeline as described in Supplementary File S1. For demo purposes, we used two genes, *PTPRG* and *P2RX7*, which were reported to be differentially expressed in microglia cell populations [34].

**Figure 2:**
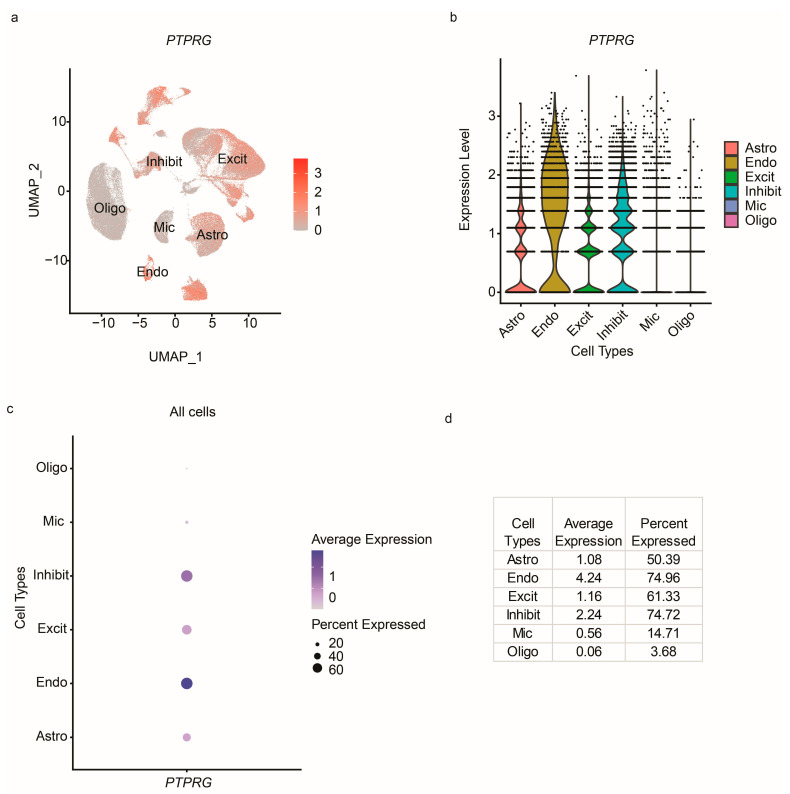
Overall expression of the *PTRPG* gene in the input single-cell dataset. (**a**) Feature plots showing the average expression of genes in the entire input dataset across cell types. (**b**) Violin plots showing the average gene expression across cell types in the entire input dataset. (**c**) Dot plot showing the average expression and percent expressed of a gene across cell types. (**d**) Table showing the average expression and percent expressed metrics across cell types. Astro: astrocytes, Endo: endothelial cells, Excit: excitatory neurons, Inhibit: inhibitory neurons, Mic: microglia, and Oligo: oligodendrocytes.

In the overall expression tab, after entering *PTPRG*, the scViewer app generated several plots and tables, as shown in Figure 2. Figure 2a,b show the expression levels of the *PTPRG* gene in endothelial cells, excitatory neurons, and inhibitory neurons (the cell types). Furthermore, the dot plots and the corresponding table, as shown in Figure 2c,d, provide the average gene expression levels and percent of cells expressing *PTPRG* across the cell types. All the results in this tab measure the expression levels of scRNA-seq data from all six combined samples (AD and Normal). The information from this tab helped to obtain an overview of the gene expression across all the samples in the input data.

**Figure 3:**
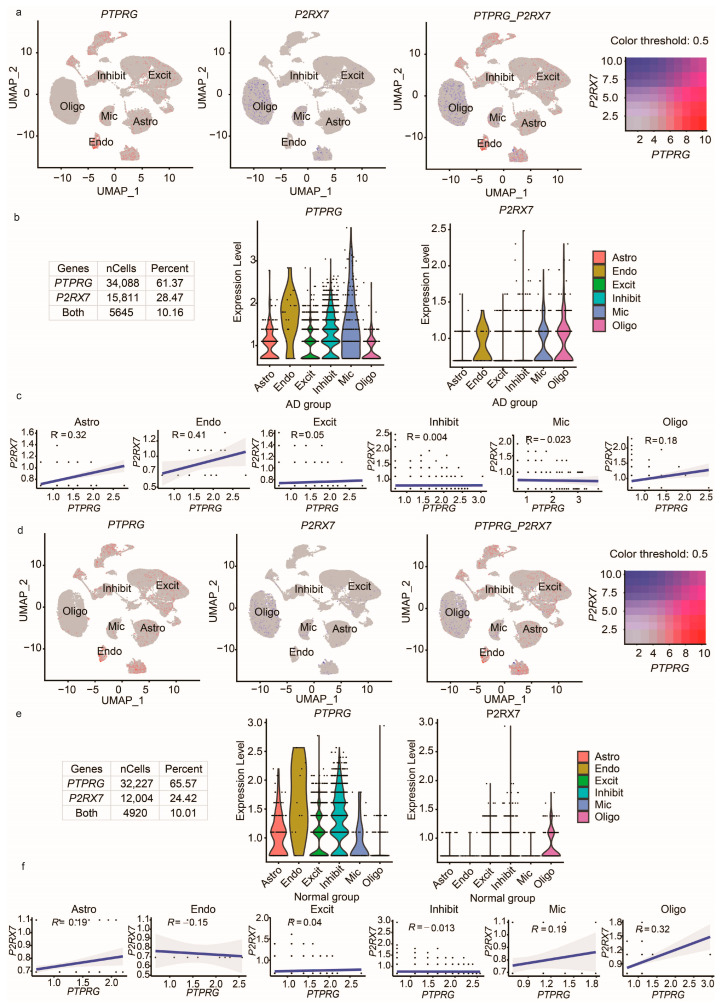
Co-expression analysis of *PTPRG* and *P2RX7* genes in AD vs. Normal population. (**a**) Feature plots showing the average co-expression of *PTPRG* and *P2RX7* genes in the AD samples. Each dot represents a cell. (**b**) Table depicting the number of cells and percent of cells expressing both *PTPRG* and *P2RX7* genes in AD samples. The violin plots show the average gene expression of *PTPRG* and *P2RX7* genes in the co-expressing cells from AD samples. Here, each dot represents a cell. (**c**) Scatter plots showing the correlation between the expressions of the two genes in the same co-expressing cells from AD samples. (**d**) Feature plots showing the average co-expression of genes in the Normal samples. (**e**) The table depicts the number of cells and percent of cells expressing both *PTPRG* and *P2RX7* genes in Normal samples. The violin plots show the average gene expression of *PTPRG* and *P2RX7* genes in the co-expressing cells from Normal samples. (**f**) Scatter plots showing the correlation between the expression of the two genes in the same co-expressing cells from Normal samples. Here, each dot represents a cell. Astro: astrocytes, Endo: endothelial cells, Excit: excitatory neurons, Inhibit: inhibitory neurons, Mic: microglia, and Oligo: oligodendrocytes.

Next, in the co-expression analysis tab, by entering *PTPRG* and *P2RX7*, the scViewer app generated three feature plots, as shown in Figure 3. The expression of *PTPRG*, *P2RX7*, and both *PTPRG* and *P2RX7* in cells from the AD samples are shown in Figure 3a. As the number of cells co-expressing these genes cannot be known from the feature plot, our app provides metrics depicting the exact number of cells co-expressing these genes (Figure 3b). We can see that there were 5645 cells co-expressing both *PTPRG* and *P2RX7* genes. The co-expressing cells were subsetted from the original data, and the violin plots (Figure 3b) show the average expression of individual genes across different cell types in the AD samples. Additionally, we show the correlation between the two genes among co-expressing cells across different cell types. We can see that the co-expression was higher in endothelial cells than in other cell types in the AD samples (Figure 3c).

Similarly, the plots in Figure 3d show the expression of *PTPRG*, *P2RX7*, and both *PTPRG* and *P2RX7* in cells from Normal samples. There were 4920 cells co-expressing both *PTPRG* and *P2RX7* genes, and the violin plots (Figure 3e) show the expression levels of these genes in different cell types from Normal samples. This comparison showed that the number of cells co-expressing the genes *PTPRG* and *P2RX7* was higher in AD samples than in Normal samples. Lastly, we measured the correlation between the two genes among co-expressing cells in Normal samples. We observed that the correlation in the astrocyte cell type was relatively higher in AD samples (R = 0.32, as shown in Figure 3c) than in Normal samples (R = 0.19, as shown in Figure 3f).

**Figure 4:**
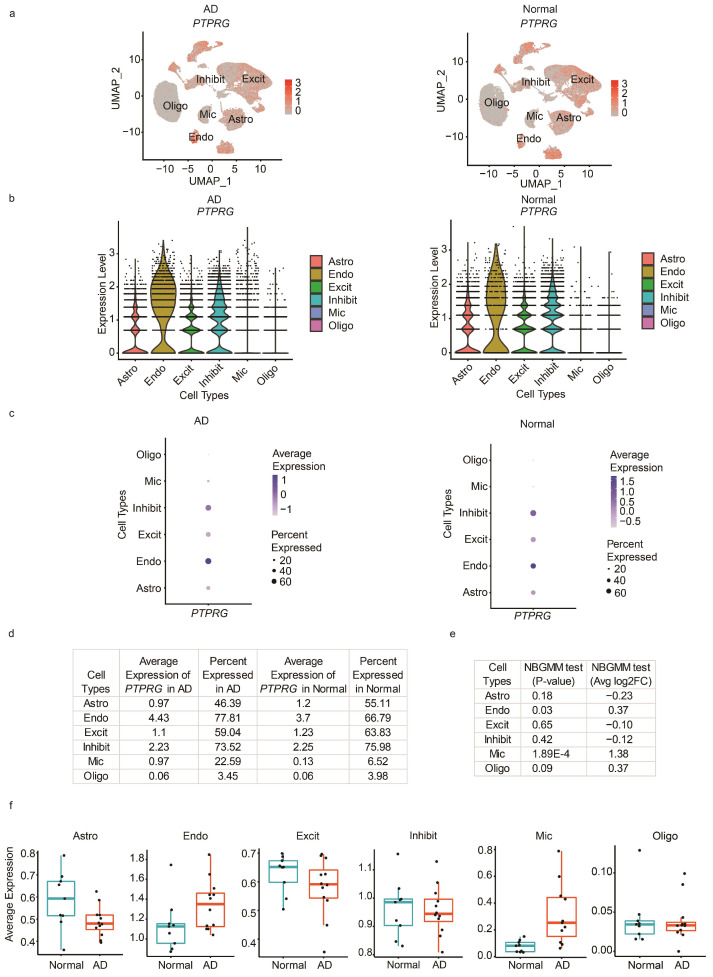
Differential expression of the *PTPRG* gene in the AD vs. Normal population. (**a**) Feature plots showing the average gene expression of the *PTRPG* gene in AD vs. Normal cells. Each dot represents a cell. (**b**) Violin plots showing a gene’s average expression of the *PTRPG* gene in the AD vs. Normal cells. Each dot represents a cell. (**c**) Dot plot showing the average expression and percent expressed of *PTRPG* gene in AD vs. Normal cells. Each dot represents a cell. (**d**) Table showing the average expression and percent expressed metrics of the *PTPRG* gene for cells from AD and Normal samples across cell types. (**e**) Table showing the differential expression for *PTPRG* gene using *p*-value and log_2_FC in all the cell types. (**f**) Boxplots showing the pseudobulk average expression of *PTPRG* gene in individual samples across different cell types. Astro: astrocytes, Endo: endothelial cells, Excit: excitatory neurons, Inhibit: inhibitory neurons, Mic: microglia, and Oligo: oligodendrocytes.

Finally, in the differential expression tab, we first entered the *PTPRG* gene, and the app provided various plots, as shown in Figure 4. The feature plots and violin plots show the expression levels of *PTPRG* in both the AD and Normal groups separately. Here, we can see that in both the AD and Normal groups, endothelial cells and inhibitory neurons had many cells expressing *PTPRG* (Figure 4b). Further, differences in the expression of *PTPRG* can be noted in the dot plot (Figure 4c), where a dot representing the average expression is visible in the microglia cell type for the AD group while it is missing in the Normal group. However, these violin and dot plots may not be helpful in understanding the difference in expression between groups with similar patterns, such as astrocytes as shown in Figure 4a–c. Therefore, our app performs the NBGMM test using nebula, as shown in Figure 4e, to show the statistical significance of the gene across cell types between the AD and Normal groups. Among all the cell types, the endothelial and microglia cell populations were statistically significant with a *p*-value of < 0.05, as shown in Figure 4e. Moreover, the highest difference in the average expression and percent expressed was observed in microglia cells (Figure 4d). The log2FC for *PTPRG* in microglia was 1.357 (Figure 4e). To visualize the average expression across individual samples, we aggregated the gene expression across the cells within an individual sample using the pseudobulk strategy, and the results are presented in boxplots (Figure 4f) showing that *PTPRG* was more abundantly expressed in AD samples than in Normal samples in microglia cells.

## 4. Discussion

Using our scViewer app, researchers can efficiently examine the gene expression patterns in scRNA-seq data across different cell types. The various expression calculations across different tabs in the app, such as overall expression, co-expression, and differential expression, are a comprehensive resource for researchers to examine a gene or a set of genes of interest in any given study with different biological or treatment conditions.

One of the key contributions of our app is the various plots and tables in the co-expression tab, which provide a detailed overview of two genes’ co-expression across different cell types. The co-expression analysis in the previously published ShinyCell [5] tool is limited as it provides only the cell count of co-expressing cells. In our app, we not only provide the cell count of co-expressing cells but also violin plots and scatter plots of these co-expressing cells, where the expression levels of two genes are shown across different cell types. These results from the co-expression tab may be particularly useful for the development of any bi-specific targets.

The other major contribution of our app is the various features provided within the differential expression tab, where the differential expression is calculated for each gene individually. This helps avoid the computational burden of performing differential expression analysis on all genes across all cells simultaneously. We provide a robust statistical test, using NBGMM from nebula, to obtain *p*-values and log_2_FC values across different cell types. These results allow the quantification of variation between cells and subjects across multiple conditions (Normal vs. disease). Several existing approaches using Wilcoxon rank sum tests, *t*-tests, and negative binomial tests do not account for subject-level variation and obtain inflated *p*-values [35,36,37,38,39]. To address this problem, subject-level variation was properly incorporated in mixed modeling by treating the subject as a random effect across different biological samples using NBGMM from nebula [24]. Testing the differential gene expression by treating each subject and not each cell as an experimental unit of interest is an important aspect of scRNA-seq analysis. Crowell et al. [37] presented a well-controlled false discovery rate (FDR) for both the pseudobulk and mixed model compared with the marker-based approach. Zimmerman et al. [36] and Squair et al. [35] showed that if the variability in gene expression across subjects is ignored it would lead to false discoveries. The major downside of using NBGMM is the computational time required to perform the analysis. Squair et al. [35] reported that the computational time required for running a mixed model was extremely high even on a relatively small dataset. We addressed this issue in our app by (1) performing gene-level analysis and (2) using nebula which was designed for fast mixed model implementation. To the best of our knowledge, these in-depth analytical features are currently not present in any of the existing tools that provide differential expression analysis [6,11]. Additionally, the gene-level analysis is a user-friendly visualization feature that is necessary to gain quick insights into multi-condition data. Although the mixed modeling method is the more accurate statistical approach for performing differential expression in single-cell data, we need to be cautious when the sample size is low (<20 samples) while interpreting the *p*-values because of the relatively inflated type I error rate [24].

As single-cell technology is evolving, many other tools are being developed to address a broad range of technical challenges in single-cell data. Lawlor et al. [40] developed an application for identifying and annotating hidden sources of variation. Interlandi et al. [41] introduced a tool for exploring cell–cell communication in scRNA-seq data. Ekiz et al. [42] developed a Shiny package to annotate cell clusters by adding scores to gene expression profiles of unknown cell clusters against mouse or human references. The unique features present in these applications are important for understanding the disease mechanisms at the single-cell level. There is a further need to evaluate these tools and incorporate them into a unified application that helps biologists to perform quicker analysis. In order to facilitate that, we plan to continuously upgrade our application and make it dynamically available by incorporating some of the exciting features and updates in single-cell biology.

In this study, we developed an app to facilitate the exploration of scRNA-seq data interactively to allow users to review cell types globally and to conduct cell-type-specific differential expression and co-expression analysis. The application is deployed within the user’s desktop web browser, enabling users to visualize the single-cell expression profiles. We aimed to provide a resource where researchers can quickly explore expression patterns and changes in an scRNA-seq study.

## Data Availability

The scripts used for data processing and analysis of the scRNA-seq data are available on GitHub (https://github.com/arpatil01/scViewer, accessed on 4 March 2023).

## Supplementary Materials

The following supporting information can be downloaded at: https://github.com/arpatil01/scViewer.
